# Supplementary material for: Modulation of Broiler Intestinal Changes Induced by Clostridium perfringens and Deoxynivalenol through Probiotic, Paraprobiotic, and Postbiotic Supplementation
Source: Toxins (Basel). 2024 Jan 14;16(1):46. doi: 10.3390/toxins16010046 (PMC10820081; doi:10.3390/toxins16010046)
Supplement: Supplementary file 1 [file toxins-16-00046-s001.zip › toxins-2759273-SI.pdf]

toxins MDPI

Supplementary Materials: Modulation of Broiler Intestinal Changes Induced by *Clostridium perfringens* and Deoxynivalenol through Probiotic, Paraprobiotic, and Postbiotic Supplementation

Marielen de Souza, Ana Angelita Sampaio Baptista, Maísa Fabiana Menck-Costa, Larissa Justino, Eduardo Micotti da Glória, Gabriel Danilo Shimizu, Camila Rodrigues Ferraz, Waldiceu A. Verri, Filip Van Immerseel and Ana Paula Frederico Rodrigues Loureiro Bracarense

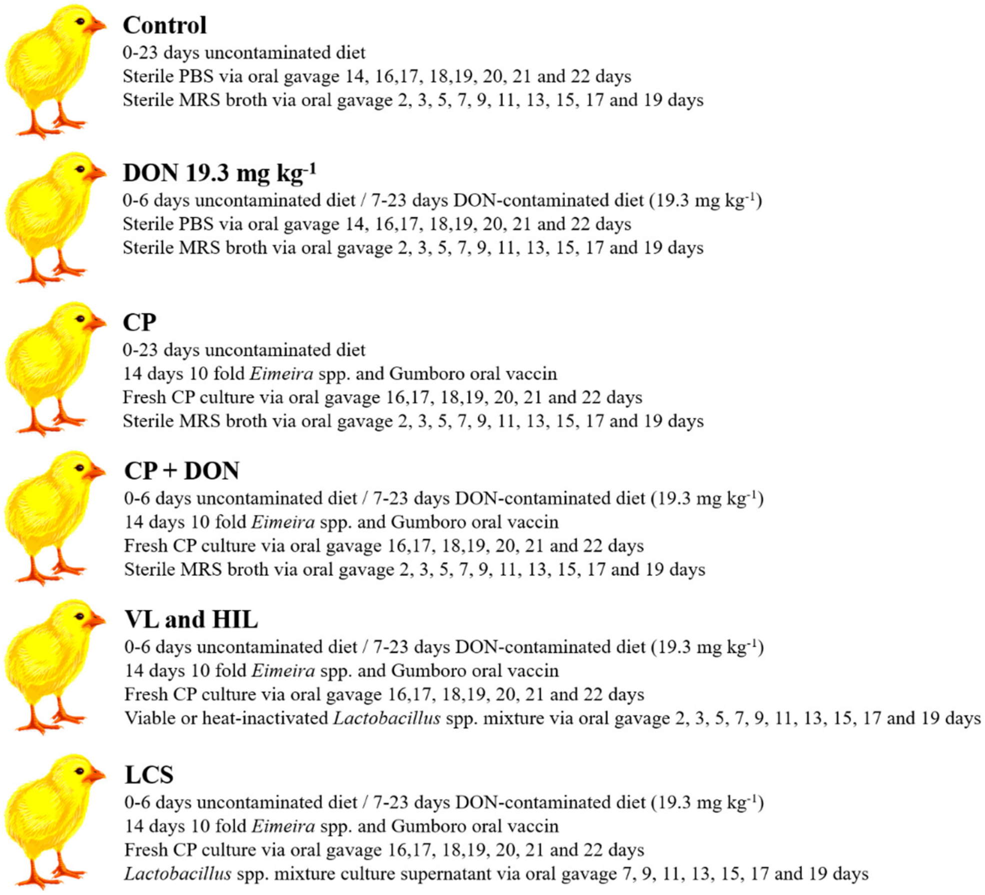

Figure S1. Experimental design. Experimental groups: Control—uncontaminated diet. DON (deoxyniva-lenol)—diet with DON 19.3 mg kg^{-1}. CP (*Clostridium perfringens*)—uncontaminated diet + *C. perfringens* challenge. CP + DON—DON 19.3 mg kg^{-1} + *C. perfringens* challenge. LV—DON 19.3 mg kg^{-1} + *C. perfringens* challenge plus viable *Lactobacillus* spp. mixture. HIL—DON 19.3 mg kg^{-1} + *C. perfringens* challenge plus heat-inactivated *Lactobacillus* spp. mixture. LCS—DON 19.3 mg kg^{-1} + *C. perfringens* challenge plus *Lactobacillus* spp. mixture culture supernatant.
